# Airflow-aligned helical nanofilament (B4) phase in topographic confinement

**DOI:** 10.1038/srep29111

**Published:** 2016-07-07

**Authors:** Min-Jun Gim, Hanim Kim, Dong Chen, Yongqiang Shen, Youngwoo Yi, Eva Korblova, David M. Walba, Noel A. Clark, Dong Ki Yoon

**Affiliations:** 1 Graduate School of Nanoscience and Technology and KINC, KAIST, Daejeon 305-701, Republic of Korea; 2 Department of Physics and Soft Materials Research Center, University of Colorado, Boulder, CO 80309, USA; 3 Institute of Process Equipment, College of Chemical and Biological Engineering, Zhejiang University, Hangzhou, 310027, China; 4 Department of Chemistry and Soft Materials Research Center, University of Colorado, Boulder, CO 80309, USA

## Abstract

We investigated a controlled helical nanofilament (HNF: B4) phase under topographic confinement with airflow that can induce a shear force and temperature gradient on the sample. The resulting orientation and ordering of the B4 phase in this combinational effort was directly investigated using microscopy. The structural freedom of the complex B7 phase, which is a higher temperature phase than the B4 phase, can result in relatively complex microscopic arrangements of HNFs compared with the B4 phase generated from the simple layer structure of the B2 phase. This interesting chiral/polar nanofilament behaviour offers new opportunities for further exploration of the exotic physical properties of the B4 phase.

Smectic phases of liquid crystals (LCs) are characterized by the layer structure built up by orientation ordering of mesogenic units^1^. Among various kinds of the layering structures, bent-core smectics form complex and interesting structures having such exotic polar and macroscopic chiral LC phases because of the translational components within the resulting smectic layers^2^,^3^,^4^,^5^,^6^,^7^. These specific behaviours are based on the strong nano-segregation of bent-core mesogenic units, leading to the formation of planar or modulated layers, producing a variety of ferro- and antiferro-electric phases. Especially, the B7 phase exhibits modulated structures due to the periodically splayed and tilted LC mesogenic units^8^ and the helical nanofilament (HNF, B4) phase is driven by local saddle splay deformation of the semi-crystalline layers^7^.

To control these complex bent-core LC structures, more sophisticated methods should be used compared with the simple nematic phase, which is easily aligned using conventional LC alignment methods^9^. The problem of orientation control of the complex B phases could be solved using the topographic surface patterning method based on lithography^10^ and the electric-field driven alignment method^11^, which has emerged as a new and powerful tool for producing single structural domains of LCs and other soft materials. This effort was extended to the organization of the complex HNF (B4) phase^12^,^13^,^14^,^15^. HNFs made by **NOBOW**, which undergoes the relatively simple B2/B3-B4 phase transition upon cooling from the isotropic phase (Fig. 1b), have been successfully controlled using the convolution method of topographic control of the confinement, shear flow, and temperature gradient^13^ or using nanoconfinement^15^. In comparison, **MHOBOW**, which contains the B7 intermediate phase upon cooling from the isotropic phase exhibits more complex morphological behaviour (Fig. 1a)^12^. Thus, this material has more structural freedom than **NOBOW** during the generation of HNFs.

Here, we discuss these specific morphological changes during the B7 to B4 phase transition based on an experimental investigation using various microscopic techniques including depolarised reflected light microscopy (DRLM) and atomic force microscopy (AFM). Understanding the formation of HNFs from the various phases can provide important ways to use chiral soft matter in potential optical applications such as second harmonic generation^16^,^17^,^18^ optical activity generation^19^ and electro-optic application^20^, as the simple nematic LC phase has been critical in present LC display industries.

## Results and Discussion

**Figure 1:**
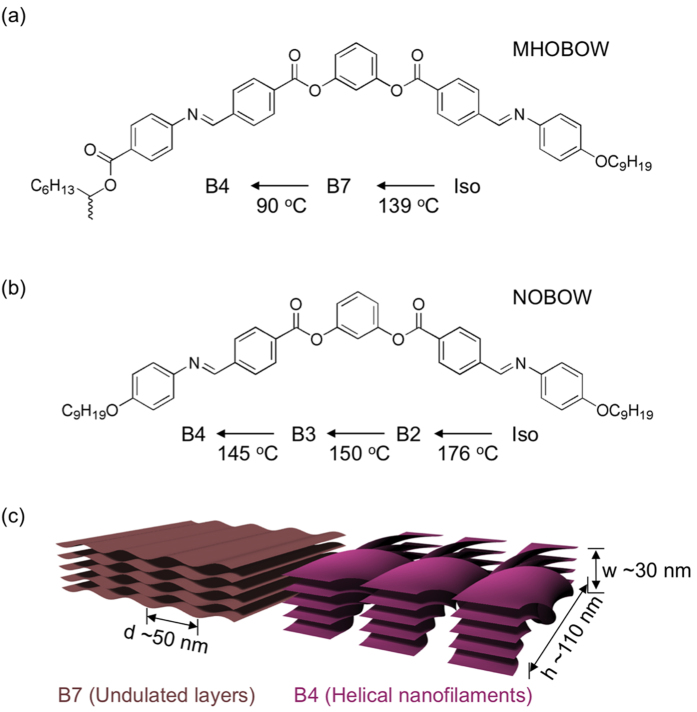
Optical structures built up by LC molecules. The molecular structure and phase sequence upon cooling from the isotropic phase of (**a**) **MHOBOW** and (**b**) **NOBOW.** (**c**) The scheme of undulated layers (B7 phase) and helical nanofilaments (B4 phase).

**Figure 2:**
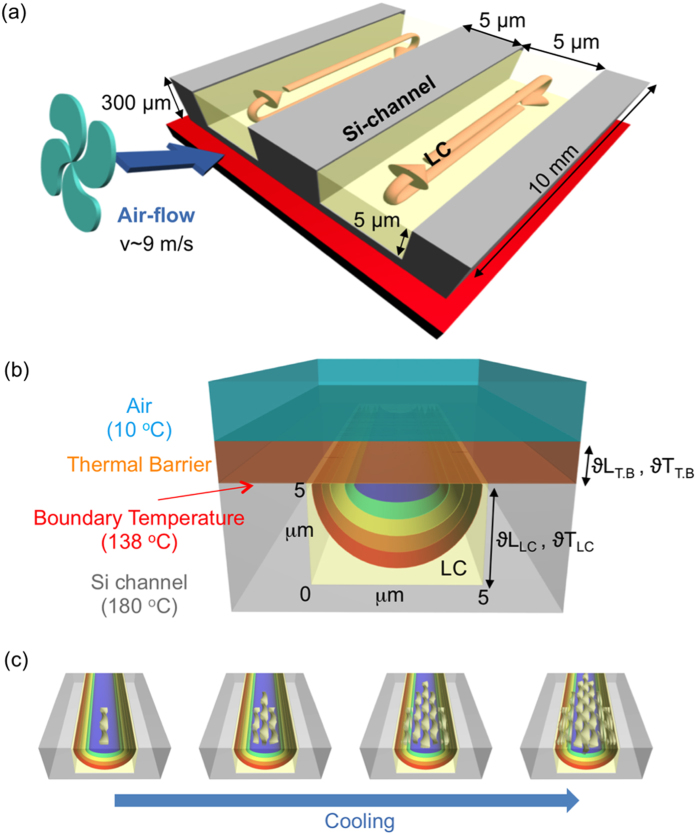
The scheme of experimental conditions. (**a**) The scheme of experimental set-up. (**b,c**) The schematic temperature gradients in the micro-channel and growing sequence with airflow. (**b**) Temperature at top-center surface of LC is just under isotropic phase (~138 °C) and at side- and bottom walls of the silicon micro-channel (~180 °C), having a big thermal gradient (∇T = 42 °C), representing schemes of boundary conditions of air, thermal barrier, and LC. (**c**) Serial perspective views of growing HNFs through the channel direction as a sequence of temperature gradient from low temperature (blue) to high temperature (red) under airflow along with the channel direction.

Upon cooling from the isotropic phase, **MHOBOW** forms the B7 phase, and the smectic layers preferentially orient normal to the Si wafer/LC and glass/LC interfaces and parallel to the LC/air interface, revealing in-plane modulation in the molecular orientation and ordering, which leads to a periodic pattern of layer undulations^21^. The layer periodicity (layer spacing of ~4 nm) and the splay modulation periodicity (splay undulation period, d, of ∼50 nm) make the B7 two-dimensionally ordered smectic layers^6^. The transition to the B4 phase from this complex B7 phase is strongly first order, with the development of more crystal-like in-plane ordering appearing in the form of HNFs. A single HNF in the B4 phase looks like a rope with width, w, of ~30 nm and half pitch, h, of ~110 nm in which 5–8 smectic layers are twisted (Fig. 1c)^7^. In the bulk phase, these rope-like twisted units self-assemble parallel together with neighboring HNFs and form “bouquet-like” splayed arrays macroscopically^7^,^13^. The growth of HNFs is more or less affected in the B7 layer structure and LC molecular interfaces. Thus, the topographic morphologies of bulk HNFs appear disordered even though each HNF is mostly grown parallel to the air/LC interface. During a cooling procedure at a rate of 0.02 °C/min, mechanical shearing was applied to the sample by the airflow (Fig. 2a). For the applied-airflow experiments, the heating stage temperature for obtaining the isotropic phase was set well above the bulk iso-B7 phase transition temperature T = 138 °C to T ~ 180 °C, where the LC in the channels is isotropic, even in the presence of airflow (Fig. 2b)^13^. The airflow results in turbulent air above the sample surface, which tends to cool the air/sample interface via thermal conduction through a thin thermal barrier layer (T.B) of air (Fig. 2b). Because the thermal conductivity of Si is much higher than that of the LC and air (λ_Si_ ~ 148 W/m°C, λ_LC_ ~ 0.2 W/m°C, and λ_air_ ~ 0.025 W/m°C)^22^, the temperature decrease of the LC surface due to the thermal flux out of the sample surface is much larger than that of the Si, in which the heat flux will be out of the surface. Thus, we can estimate a temperature gradient from the LC surface without or with airflow using the following heat flux equation and boundary conditions:


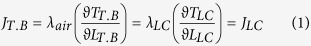


Here, J is the heat flux, ϑT is the temperature difference between the two media, λ is the thermal conductivity of the media, and ϑL is the distance over which the temperature drop is achieved. With no airflow, we can determine the temperature gradient in the LC at its surface using ϑL_T.B_ ~ 1 cm and the boundary conditions λ_air_ = 0.025 W/m °C, λ_LC_ = 0.2 W/m°C, ϑT_T.B_ = 138 − 10 = 128 °C, and ϑL_LC_ = 5 × 10^−6^ m in equations (1) and (2).


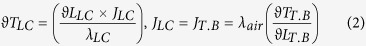


Thus, the temperature gradient (∇T) in the LC at its surface is ~0.0016 °C/μm. With airflow, we can estimate the boundary layer thickness using equation (3):


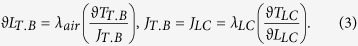


Here, the boundary layer thickness ϑL_T.B_ is ~1.9 μm when ϑT_LC_ = 180–138 = 42 °C, and the other conditions are same with equation (2). Then, we can determine the thermal gradient in the LC (∇T ~ 8.4 °C/μm) using the same calculation as that shown in equation (2). Thus, we can estimate that the LC surface temperature is ~42 °C cooler than that of Si, with the contours shown in Fig. 2c. The two-dimensional (2D) temperature distribution in the channel (Fig. 2c) has the lowest LC temperature along the LC centerline at the air/LC interface^13^.

**Figure 3:**
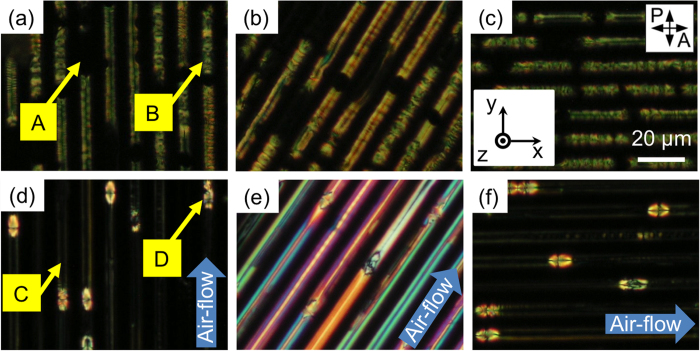
Optical textures of B4 phase with/without airflow. (**a–c**) The B4 phase of **MHOBOW** in the channels shows two types of optical structures of mostly dark (A) and bright rectangular (B) domains before applying airflow. (**d–f**) The textures of B4 phase with airflow, in which they also have two kinds of domains of dark (C) and extended hexagonal (D) ones.

**Figure 4:**
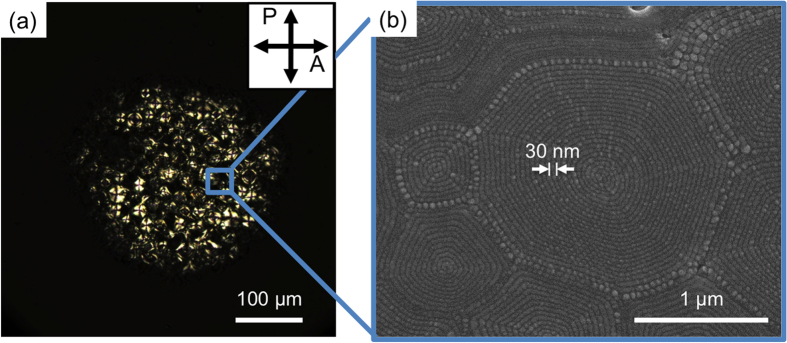
Morphology of HNFs in a bulk sample. (**a**) DRLM image of circular domains of B4 in a bulk sample. (**b**) SEM image of the circular domains. HNFs form spiral patterns that result from nucleation and growth. Pattern period (~30 nm) is corresponding to width of a single HNF in Fig. 1c.

To compare the airflow effect, the simply confined B4 phase of the **MHOBOW** sample in the microchannels was verified. First, DRLM was used to probe the resulting optical texture and ordering of the HNFs during rotation of the sample to determine the optical anisotropic characteristic of the sample at room temperature (Fig. 3a–c). The B4 phase of **MHOBOW** in the microchannel forms two types of optical morphologies of just dark **(A)** and bright rectangular domains **(B)** along with the microchannels (Fig. 3a–c). The dark region **(A)** of the confined B4 phase remained dark, whereas birefringence of the rectangular domains **(B)** of the B4 phase changed from green to orange during rotation of the sample. This phenomenon was demonstrated in a previous paper, in which the optical anisotropy of HNFs produced using **NOBOW** can be cancelled because of the continuously changing optical axis of the mesogenic units in HNFs^13^. Even though the rectangular optical textures of **MHOBOW** have not been observed before, the rectangular shape was determined to be derived from the circular surface domain in the B7 phase (Fig. 4a,b)^12^.

The airflow-aligned sample in the microchannels exhibited a quite different morphology compared with those mentioned above. The DRLM images revealed diverse colours when the sample was placed at 45° to the cross-polarisers (Fig. 3e), though the dark regions (marked **C** in Fig. 3d) still remained dark when the channel direction was parallel or perpendicular to the crossed polarisers (Fig. 3d,f). This finding indicates that the optical axis of **MHOBOW** molecules is well aligned even after the phase transition to the B4 phase. In the unaligned sample (Fig. 3a–c), the birefringence is cancelled out because of the molecules in the twisted layers of HNFs, whereas the molecules are somewhat well-aligned in the airflow-induced case. This tendency can be also observed in the rectangular domains, which look similar to the extended texture of **(B)** after applying airflow on the sample **(D)**; in addition, the population of this type of domain with a pseudo-Maltese cross pattern is significantly decreased.

**Figure 5:**
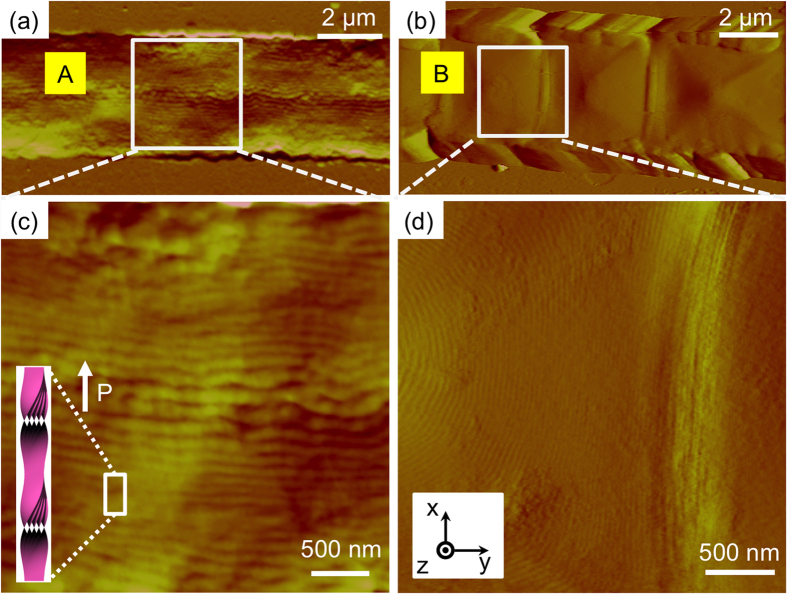
AFM images of confined B4 phase of MHOBOW in the micro-channel. (**a,c**) Dark region (A) in Fig. 3a shows aligned line structures (h~110 nm) parallel to channel direction, which means HNFs are aligned perpendicular to the channel direction. (**b,d**) Rectangular region of (B) in Fig. 3a shows a spiral configuration.

To see the effect of airflow on the generation of HNFs at the nanoscale, AFM experiments were performed. First, the HNFs before shearing the sample by airflow were probed (Fig. 5). The two types of B4 structures of **MHOBOW** in the channels were directly examined, revealing elongated line patterns **(A)** and periodic rectangular patterns **(B)** along with the microchannel, which is consistent with the findings observed in the DRLM images (Fig. 3a–c). The line patterns in the **A**-type area are mostly aligned through the channels; however, there are some undulations, which can be interpreted as HNFs aligned perpendicular to the channel walls. The distance between lines is ~110 nm, which coincides with the h value shown in Fig. 1c. This result originated from the nucleation and growth from the side walls of the microchannels^13^.

The **B**-type of periodic rectangular patterns was observed in the AFM micrographs, which is consistent with the DRLM images (Fig. 3a–c). The **B**-type morphology contains small-featured line structures (~35 nm) in the enlarged AFM image (Fig. 5d), and the distance between lines decreases upon approaching the boundary area of the rectangular patterns in which this distance corresponds to the width of the HNFs (w ~ 30 nm). This finding indicates that the HNFs are aligned with spiral configuration in the rectangular patterns (Fig. 4b)^12^. In general, during the phase transition from polar smectic phases to the B4 phase, the helical axis of the HNF guided by the polar directors of LC molecules at the higher-temperature phase forms LC phases, e.g., B2 and B7 phases^7^. Although it is difficult to consider that the HNFs are highly bent in the macroscopic rectangular patterns because of their crystal-like characteristics, the high degree of freedom of smectics in the B7 phase enables the formation of the spiral configuration of HNFs (Fig. 4b)^12^. This behaviour differs significantly compared with the B4 phase having a B2 phase made by **NOBOW** at the higher temperature (Fig. 1b)^13^, in which the focal conic domains (circular domains) of the B4 phase were generated by following the smectic layers of the B2 phase. The different phase sequence and degrees of freedom of former (or higher-temperature) phases induce the different orientation of HNFs after the phase transition to the B4 phase.

**Figure 6:**
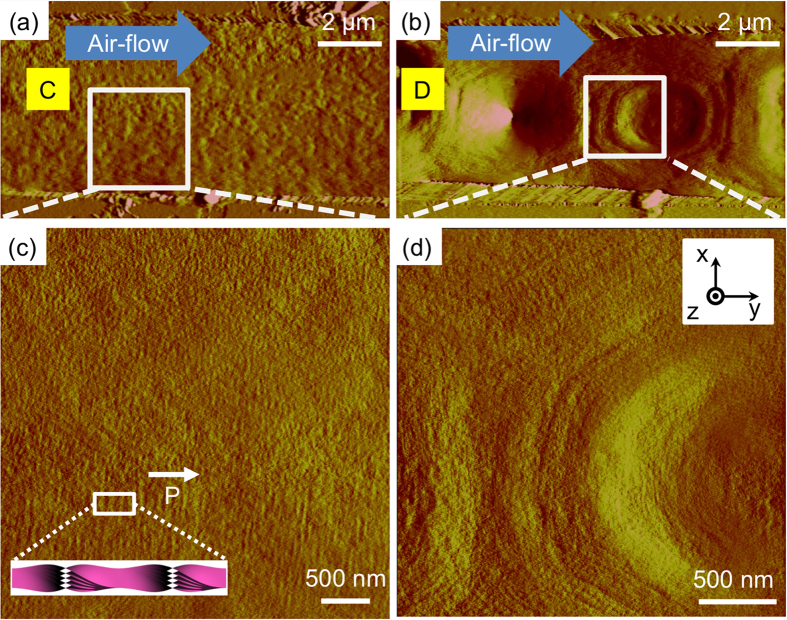
AFM images of B4 phase in the microchannel after applying airflow. (**a,c**) Dark region (C) in Fig. 3d shows aligned line structures (h ~ 90 nm) perpendicular to the airflow direction, meaning the HNFs are aligned along with the channel direction. (**b,d**) Extended hexagonal regions (D) in Fig. 3d also show dim surface structures.

The airflow-induced B4 phase was also examined. Notably, the surface morphology of the **C** pattern (Fig. 6a,c) was very smooth compared with that of the original sample (Fig. 5a,c). Very dim line patterns exhibit ~95 nm periodicity, and sometimes, it is too dim to clearly see the exact morphology. The direction of line patterns changed to be perpendicular to the channel direction, which indicates that most of the HNFs are aligned along with the channel, although deformed structures were generated (Fig. 6c). Here, it can be considered that the mesogenic units themselves are aligned through the airflow direction; however, the HNFs of **MHOBOW** are deformed. These two distinctive characteristics can explain the colourful birefringence in the DRLM images and the different tendency compared with the regularly aligned HNFs in the previous study^13^. Thus, the topographically dim surface structures with unidirectional aligned mesogenic units can be considered deformed HNFs, in which a relatively weak molecular arrangement resulting from the relatively weak interaction between **MHOBOW** molecules exists. This type of higher-temperature-dependent weak bond was also observed in nanoconfined HNFs, in which there are two types of HNFs that have B2 smectic layers **(NOBOW)** and B1 columns (W618 and W513), and the HNFs with the B1 phase at higher temperature could be easily deformed because of the modulated characteristics induced by the B1 phase^23^,^24^.

In the **D**-type domains, the rectangular structures appearing without airflow were also deformed to generate asymmetric hexagonal domains extended through the direction of airflow (Fig. 6b,d), and a similar dim morphology is observed in Fig. 6a,b. During the phase transition from the B7 to B4 phase, the shearing force induced by the airflow could interrupt the generation of HNFs from the B7 smectic phase even though the airflow helped the LC molecules to align. Thus, we observed uniform optical textures with large birefringence using DRLM (Fig. 3e) and the non-periodic morphology using AFM for the B4 phase (Fig. 6c,d).

Based on these macroscopic and microscopic observations, the importance of the higher-temperature phases for the generation of HNFs is apparent. Their importance is strongly related to the different premade structural characteristics of the higher phase compared with those of the HNFs, e.g., B7 and B2. In the B2 smectic layers, the layer polarisation and chirality of the B2 phase can be racemic or homo-chiral; however, the bent-core molecules are definitely closely packed with uniform polar and tilt senses. In contrast, the in-plane layers of the B7 phase are spatially modulated with a specific periodicity, in which the polar and tilt directors of the molecules are continuously changed, resulting in various types of characteristic textures such as spiral textures consisting of smectic filaments and myelin-like, accordion-like, checker-board-like, banana-leaf-like, and circular domain textures^6^,^25^. Thus, the molecules and superstructures of HNFs of **MHOBOW** can be more frustrated than those of **NOBOW** in our experimental system. These orientation behaviours can be clarified in two ways. Once **MHOBOW** molecules are self-organized into HNFs, the HNFs can undergo large structural deformation (e.g., spiral patterns), and the orientation of the HNFs is easily affected by an external force because of the weaker interaction of molecules in B7/B4 phases compared with HNFs of **NOBOW**. However, if there are excessive external stimuli applied to the LC molecules during the phase transition from B7 to B4, the mesogenic units cannot be well-organized to form HNFs and microscopically respond to the stimuli, resulting in the uniaxially oriented LC molecules along the direction of the shearing force, as depicted in the DRLM and AFM micrographs.

## Conclusions

We investigated the physical behaviour of complex chiral crystals (HNFs) of the B4 phase, built up **MHOBOW** with a high degree of freedom, under convolution methods of shearing force by airflow and topographic confinement in silicon microchannels. Various macroscopic and microscopic visualizations revealed that the HNFs of **MHOBOW** molecules have more flexible structural characteristics than HNFs made using **NOWBOW** molecules, implying the self-organization into HNFs can easily be disturbed and controlled by external forces. This result is of use in further exploration of the exotic physical properties of B4 phases accompanying the design of the soft building blocks to form chiral structures.

## Methods

Sample and preparation of confined nanofilaments in the channels: A bent-core molecule exhibiting the B4 phase was prepared as reported previously, forming the B7 intermediate phase upon cooling from an isotropic temperature (Fig. 1a)^7^. The bent-shaped molecular structure composed of aromatics and flexible alkyl tails exhibited smectic layering of spontaneously polar order and tilt to reveal twisted layers of the B4 phase from undulated layers of the B7 phase (Fig. 1c). The rectangular microchannels of the Si wafer were prepared using photolithography and reactive ion etching techniques^10^,^13^,^14^,^21^. These microchannels had a square cross section with a depth and width of 5 μm and length of 10 mm (the total thickness of the Si substrate was 300 μm) (Fig. 2a). The microchannels were chemically cleaned for planar anchoring by immersion in a mixture of dimethylformamide (DMF) and methanol to remove organic–inorganic impurities, followed by rinsing several times with deionized water. Materials were poured on the edge of the channels and filled by capillary force at isotropic temperature using a heating stage (INSTEC HCS410) and a controller (INSTEC STC 200) and then cooled down to room temperature at a rate of 0.02 °C/min. Upon cooling from the isotropic temperature, airflow was supplied by a 4-mm-inner-diameter Cu pipe, in which the temperature was approximately 10 °C, and the flux of air was controlled by a regulator at a rate of 15 ft^3^/min (0.42 m^3^/h), yielding a flow velocity of approximately 9 m/s across the surface (Fig. 2a).

### Microscopy

The optical textures of the samples in the microchannels of the B4 phase were examined using DRLM (Nikon Eclipse E400 POL) at room temperature. The surface topography of the sample was examined under ambient conditions using an AFM (Nanoscope III: Veeco Instruments, Santa Barbara, CA). Contact mode AFM with Si_3_N_4_ cantilevers having a small spring constant of 0.06N/m was used to minimize unexpected deformation of the sample.

## Additional Information

**How to cite this article**: Gim, M.-J. *et al*. Airflow-aligned helical nanofilament (B4) phase in topographic confinement. *Sci. Rep.* **6**, 29111; doi: 10.1038/srep29111 (2016).
